# The Genetic Variation of Lactase Persistence Alleles in Sudan and South Sudan

**DOI:** 10.1093/gbe/evab065

**Published:** 2021-03-24

**Authors:** Nina Hollfelder, Hiba Babiker, Lena Granehäll, Carina M Schlebusch, Mattias Jakobsson

**Affiliations:** 1 Human Evolution, Department of Organismal Biology, Uppsala University, Sweden; 2 Department of Linguistic and Cultural Evolution, Max Planck Institute for the Science of Human History, Jena, Germany; 3 Institute for Mummy Studies Eurac Research, Bolzano, Italy; 4 SciLifeLab, Uppsala University, Sweden; 5 Palaeo-Research Institute, University of Johannesburg, Auckland Park, South Africa

**Keywords:** lactase persistence, Northeast Africa, Sudan, South Sudan, genetic diversity, selection

## Abstract

Lactase persistence (LP) is a well-studied example of a Mendelian trait under selection in some human groups due to gene-culture coevolution. We investigated the frequencies of genetic variants linked to LP in Sudanese and South Sudanese populations. These populations have diverse subsistence patterns, and some are dependent on milk to various extents, not only from cows but also from other livestock such as camels and goats. We sequenced a 316-bp region involved in regulating the expression of the *LCT* gene on chromosome 2, which encompasses five polymorphisms that have been associated with LP. Pastoralist populations showed a higher frequency of LP-associated alleles compared with nonpastoralist groups, hinting at positive selection also among northeast African pastoralists. Among the LP variants, the -14009:G variant occurs at the highest frequency among the investigated populations, followed by the -13915:G variant, which is likely of Middle Eastern origin, consistent with Middle Eastern gene flow to the Sudanese populations. There was no incidence of the “East African” LP allele (-14010:C) in the Sudanese and South Sudanese groups, and only one heterozygous individual for the “European” LP allele (-13910:T), suggesting limited recent admixture from these geographic regions. The Beja population of the Beni Amer show three different LP variants at substantial and similar levels, resulting in one of the greatest aggregation of LP variants among all populations across the world.

**Significance**

Africa displays a large variation in lactase persistence alleles with all known variants found on the continent, but studies focusing on lactase persistence in Africa have rarely concentrated on all five alleles and many African regions are still understudied. Our study investigates all currently known lactase persistence-associated variants in 18 populations from Sudan and South Sudan. We find among the highest frequencies of putative lactose digesters worldwide in Sudanese pastoralists. In contrast to European populations, where the high level of lactose digesters is caused by one variant, populations in Sudan carry multiple alleles associated with lactase persistence. We find a diversity of African lactase persistence alleles and suggest that gene flow shaped the diverse lactase persistence landscape in Northeast Africa.

## Introduction

Lactase persistence (LP) is the ability to digest the milk sugar, lactose, at an adult age. The phenotype is associated with several single nucleotide polymorphisms (SNPs) that are located 13.9 kb upstream of the lactase gene (*LCT*) in an associated enhancer element. Currently, we know of at least five variants that are clearly associated with the LP phenotype (Enattah et al. 2002; Ingram et al. 2007, 2009; Tishkoff et al. 2007). The best-known case is the -13910:C>T polymorphism (rs4988235), which is strongly associated with LP in populations of European ancestry (Enattah et al. 2002) and has been under strong recent selection, likely coevolving with dairy farming (Bersaglieri et al. 2004).

The LP phenotype has been found at greater frequencies in milk-drinking pastoralist populations than nonpastoralist populations (Holden and Mace 1997; Tishkoff et al. 2007; Itan et al. 2010; Gerbault et al. 2011). However, LP occurs in populations that do not carry the derived -13910:T allele, specifically in the Middle East and Eastern Africa. Therefore, the thoroughly investigated -13910:C>T polymorphism is not the causal variant in these populations (Mulcare et al. 2004; Myles et al. 2005). Other SNPs have been identified to be the putative causal variants in these regions: -13907:C>G (rs41525747) in Ethiopia and Saudi Arabia, -13915:T>G (rs41380347) in Saudi Arabia, -14009:T>G (rs869051967) in African Arab groups, and -14010:G>C (rs145946881) in Kenya and Tanzania (Ingram et al. 2007, 2009; Tishkoff et al. 2007; Jones et al. 2013; Priehodová et al. 2014; Ranciaro et al. 2014; Liebert et al. 2016). These polymorphisms have been shown to increase *LCT* promoter expression in vitro (Ingram et al. 2007; Tishkoff et al. 2007; Enattah et al. 2008; Jensen et al. 2011; Olds et al. 2011; Jones et al. 2013; Liebert et al. 2016), and the -13910:C>T variant was recently identified as the putative causal variant for LP in a genome-wide association study (GWAS) study in the Fulani population of the African Sahel/Savannah belt (Vicente et al. 2019). There is evidence for a selective sweep on -14010:G>C (Tishkoff et al. 2007) that shows a stronger selection coefficient in the Massai in Kinyawa, Kenya (MKK) than the allele -13910:T shows in the European (CEU) population (Altshuler et al. 2010; Schlebusch et al. 2013), pointing to a strong increase in fitness for LP individuals in African pastoralist populations.

LP-associated SNPs have been reported in Northeast Africa (Ingram et al. 2007; Tishkoff et al. 2007; Enattah et al. 2008; Hassan et al. 2016) and there is linguistic and archaeological evidence that cow-herding has been practiced in northeast Africa for at least 4,000 years (Ehret 1979; Smith 1992). The development of farming in northeast Africa depended on the climatic conditions. Although the wetter conditions along the Nile allowed for crop farming and settlement, pastoralism with a seminomadic lifestyle was developed in the drier Savannah/Sahel regions (Haaland R and Haaland G, 2013). The pastoralist Beja populations of Sudan have been shown to have a high prevalence of LP (Bayoumi et al. 1981; Tishkoff et al. 2007) and moderately high frequencies of LP-associated alleles compared with neighboring populations, which could have arisen due to a selection event (Ranciaro et al. 2014). The Nilotic populations of current-day South Sudan are dairy-consuming pastoralists, which have been shown to be lactase persistent in low frequencies (Bayoumi et al. 1981, 1982; Tishkoff et al. 2007), but no alleles associated with LP have this far been found (Tishkoff et al. 2007; Hassan et al. 2016).

To deepen our understanding of LP in Northeast Africa and the associated variants, we sequenced a 316-bp region spanning all known SNPs associated with LP in 221 individuals from 18 Sudanese and South Sudanese (SASS) populations. Combining this data with previously published high-density genome-wide genotyping data of the same individuals (Hollfelder et al. 2017) and comparative populations genotyped in the 1000 Genomes Project Consortium (2015), we were able to investigate the allele frequencies of the LP-associated SNPs and their haplotype backgrounds and to scan for signals of selection.

## Results and Discussion

### Allele Frequencies of LP-Associated Alleles

In total, we identified nine different polymorphisms in this study (table 1). We detected four (-13907:G, -13910:T, -13915:G, and -14009:G) of the five LP-associated alleles and their frequencies per population are shown in table 2. None of the LP-associated SNPs are significantly deviating from Hardy–Weinberg equilibrium in the investigated SASS populations.

**Table 1:** SNPs Identified on the Targeted Sequences

Locus	Alleles	SNP ID	Bp Pos. (hg19)	Derived Allele Count
**-13907**	C>G	rs41525747	136608643	11
**-13910**	C>T	rs4988235	136608646	1
-13913	C>T	rs41456145	136608649	2
**-13915**	T>G	rs41380347	136608651	27
**-14009**	T>G	rs869051967	136608745	26
**-14010**	G>A	rs145946881	136608746	1
-14011	G>A	rs4988233	136608747	1
-14107	C>T	rs574071884	136608843	1
-14108	G>A	rs56150605	136608844	1

Note.—Loci previously identified to harbor LP-alleles are highlighted in bold. The last column shows the allele count of the derived allele in the SASS data set of 203 individuals. The allele -14010:A is not the derived variant previously associated with LP and has not been added to the calculation of predicted lactase-persistence phenotype.

**Table 2:** Population Information and Allele Frequencies of Alleles at Positions Associated with LP

Population	Collective Group Name	*n*	Language Family	Traditional Subsistence Pattern	-13907:G ± SE	-13910:T ± SE	-13915:G ± SE	-14009:G ± SE	-14010:C ± SE	Predicted LP Phenotype (%)
Bataheen	Sudanese Arab	9	Afro-Asiatic	Agro-pastoralist	0	0	0.278 ± 0.106	0.167 ± 0.088	0	66.7
Gaalien	Sudanese Arab	12	Afro-Asiatic	Agriculturalist	0	0.042 ± 0.041	0.042 ± 0.041	0.125 ± 0.068	0	41.7
Messiria	Sudanese Arab	8	Afro-Asiatic	Pastoralist	0	0	0.125 ± 0.083	0	0	12.5
Shaigia	Sudanese Arab	12	Afro-Asiatic	Agriculturalist	0.042 ± 0.041	0	0.084 ± 0.056	0.125 ± 0.068	0	41.7
Copt	Copt	11	Afro-Asiatic	Agriculturalist	0	0	0	0	0	0
Hausa	Hausa	5	Afro-Asiatic	Agriculturalist	0	0	0	0	0	0
Beni Amer	Beja	16	Afro-Asiatic	Pastoralist	0.25 ± 0.077	0	0.25 ± 0.077	0.281 ± 0.080	0	87.5
Hadendowa	Beja	9	Afro-Asiatic	Pastoralist	0.056 ± 0.054	0	0.222 ± 0.098	0.333 ± 0.111	0	88.9
Danagla	Nubian	12	Nilo-Saharan	Agriculturalist	0.042 ± 0.041	0	0.042 ± 0.041	0.042 ± 0.041	0	25
Halfawieen	Nubian	9	Nilo-Saharan	Agriculturalist	0	0	0.111 ± 0.074	0	0	22.2
Mahas	Nubian	14	Nilo-Saharan	Agriculturalist	0	0	0	0.036 ± 0.035	0	7.1
Baria	Nilotic	5	Nilo-Saharan	Agro-pastoralist	0	0	0	0	0	0
Dinka	Nilotic	14	Nilo-Saharan	Agro-pastoralist	0	0	0	0	0	0
Nuer	Nilotic	15	Nilo-Saharan	Agro-pastoralist	0	0	0	0	0	0
Shilluk	Nilotic	16	Nilo-Saharan	Agro-pastoralist	0	0	0.031 ± 0.031	0	0	6.3
Gemar	Gemar	5	Nilo-Saharan	Agro-pastoralist	0	0	0.1 ± 0.095	0	0	20
Zaghawa	Zaghawa	15	Nilo-Saharan	Agro-pastoralist	0	0	0	0	0	0
Nuba	Nuba	16	Nilo-Saharan and Niger-Congo	Agriculturalist and Agro-pastoralist	0	0	0	0	0	0
Scandinavians (Bersaglieri et al. 2004)	Scandinavian		Indo-European	Agro-pastoralist		0.815				
Saudi (Imtiaz et al. 2007)	Arab		Afro-Asiatic	Agro-pastoralist			0.594			

Note.—Population refers to the self-assigned identity, whereas the collective group name refers to a larger unit connecting populations based on one or more shared traits, such as history and culture, language, religion, geographic location, ancestry, and others.

The LP-associated alleles -13907:G, -13915:G, and -14009:G appear in frequencies up to 0.34 in Sudan, mainly in Sudanese Arab, Nubian, and Beja populations (table 2). The most commonly occurring LP-associated allele found in the investigated populations is -13915:G, followed by -14009:G (table 1). The allele -14009:G has previously been found in the Beja populations of Sudan as well as African Arab groups and populations of the Middle East and East Africa (Ingram et al. 2009; Jones et al. 2013, 2015; Priehodová et al. 2014; Ranciaro et al. 2014; Liebert et al. 2016), whereas -13907:G was found primarily in populations of Sudan and East Africa (Ingram et al. 2007; Tishkoff et al. 2007; Jones et al. 2013; Ranciaro et al. 2014). The LP-associated allele -13915:G has previously been found on the Arabian Peninsula, where it likely originated (Enattah et al. 2008; Priehodová et al. 2017). It is also present in East Africa due to gene flow (Imtiaz et al. 2007; Ingram et al. 2007, 2009; Tishkoff et al. 2007; Enattah et al. 2008; Priehodová et al. 2017). In this study, the allele frequency of -13915:G correlates significantly (ρ* *= 0.588, *P* = 0.010) with the Middle Eastern admixture proportions of the investigated populations that carry the allele (supplementary fig. S1, Supplementary Material online).

The allele associated with LP in Europeans, -13910:T, was almost completely absent from the investigated populations, except for one heterozygous Gaalien individual (tables 1 and 2). The -13910:T allele has previously been detected in African populations, as a result of European gene flow, and has also been reported to occur in populations of Sudan (in low frequencies) (Enattah et al. 2007; Ingram et al. 2007; Lokki et al. 2011; Ranciaro et al. 2014; Jones et al. 2015; Hassan et al. 2016; Vicente et al. 2019). The LP-associated allele -14010:C was absent in the SASS populations. This allele occurs most commonly in the Afro-Asiatic and Nilo-Saharan pastoralist populations of East Africa (Tishkoff et al. 2007; Wagh et al. 2012; Schlebusch et al. 2013). One Bataheen individual carried a derived adenine allele at this position. This allele has been detected previously in various populations at very low frequencies and has not been associated with LP.

#### The Beja Populations

**Fig. 1:**
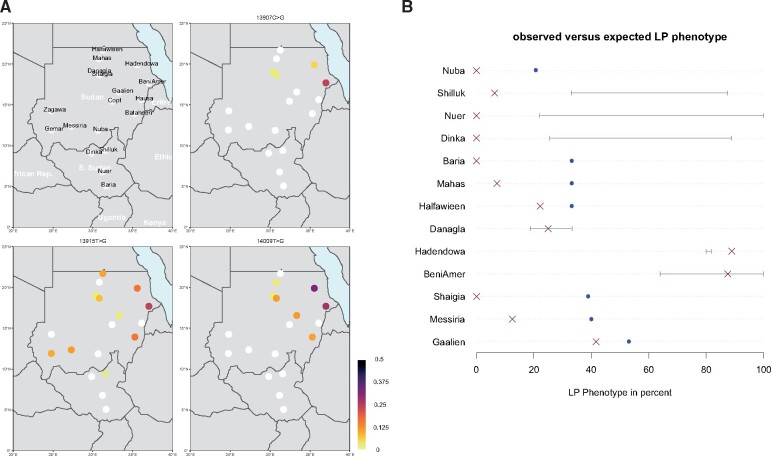
Overview of LP-associated allele frequency and LP phenotype distribution in Sudan and South Sudan. (*A*) Populations and allele frequency distribution of the three LP-associated alleles found in multiple SASS populations. The gray dot underlying the population names shows the geographic midpoint of the populations sampled in this study. A distribution of these alleles (including -13010:T and -14010:C) in Africa can be seen in supplementary figure S7, Supplementary Material online. The map shown here does not reflect the disputed border lines between Sudan, Egypt, and South Sudan. (*B*) Comparison of observed versus expected LP phenotype. Estimates based on the genetic data are shown in red, observed values from literatures are shown in blue. A range is reported if there are several observations, otherwise, a point estimate is shown as a dot. Only populations with reported observed LP phenotypes were included in the comparison. Some observations stem from larger population groupings, for example, the observed value for Baria was reported for Nilotic people.

The two Beja clans, the Beni Amer and the Hadendowa, show the highest frequencies of LP-associated alleles among the investigated populations (fig. 1*A*). All three alleles have previously been observed in the Beja populations (Tishkoff et al. 2007; Ranciaro et al. 2014; Jones et al. 2015; Hassan et al. 2016;). The Beja display the highest allele frequencies for the derived alleles at positions -13907 and -14009, as has been reported previously (Tishkoff et al. 2007; Ranciaro et al. 2014). The Beni Amer show similar levels of allele frequency for -13907:G, -13915:G, and -14009:G (0.25–0.281 [0.10–0.437]). The Hadendowa show more variation of the derived allele frequencies with higher occurrence of -14009:G (0.333 [0.116–0.551]) and a lower frequency of -13907:G (0.056 [0–0.16]). However, the -13907:G variant was previously reported at higher frequencies in the Hadendowa than observed here (Ranciaro et al. 2014). Although -13907:G is fairly common in the Beja, other Sudanese populations carry this allele only in low frequency. The only population carrying similarly high levels of -13907:G is Ethiopian Afar (Jones et al. 2015), hinting at a connection between these two Cushitic-speaking populations. The comparatively high allele frequencies of LP-associated alleles lead to the highest prediction of LP-phenotype of close to 90% in the Beja populations (table 2). This is in agreement with earlier studies that have registered the LP-phenotype to be 64–100% in the Beni Amer and 82% in the Hadendowa (fig. 1*B*) (Bayoumi et al. 1981, 1982; Holden and Mace 1997; Tishkoff et al. 2007).

#### The Sudanese Arab Populations

The genetic differentiation between the Arabs of central/north Sudan and the Messiria of southwest Sudan (Babiker et al. 2011; Hollfelder et al. 2017) is also seen in the LP-associated allele frequencies. The derived allele for -14009 is found in the Bataheen, Gaalien, and Shaigia at 0.125–0.167 frequency [0–0.339] but not in the Messiria (site specific $F_{\text{ST}}^{\text{Messiria},\;X}$= 0.062–0.114, where *X* is one of the other Sudanese Arab populations). The Messiria are part of the Baggara Arabs, a collective term for nomadic, dairy-farming pastoralist tribes of Kordofan (Bayoumi et al. 1981). Priehodová et al. (2017) hypothesized that there were two directions of Middle Eastern gene flow into the Sudan, one entered along the Nile giving rise to the Arab populations that reside along the Nile, whereas the other followed the Mediterranean coast and then turned south toward Lake Chad and entered Sudan from the west, forming the Baggara Arabs. This is supported by the genetic differentiation between the Messiria and the Arabs of central/north Sudan, their genetic proximity to their neighboring population (Hollfelder et al. 2017), and could potentially explain the absence of the LP-associated alleles other than -13915:G in the Messiria. Alternatively, through the lower levels of admixture seen in the Messiria (Hollfelder et al. 2017), only -13915:G might have been established in this population. The allele -13907:G was only found at low frequency (<0.05) in the Shaigia Arab population but it has previously been observed in low frequency in other sedentary Arab populations of Sudan (Ingram et al. 2007; Enattah et al. 2008; Ranciaro et al. 2014; Hassan et al. 2016).

#### The Nubian Populations

The Nubians (Danagla, Halfawieen, and Mahas) show low frequencies of the LP-associated alleles. The Danagla have three individuals with one heterozygous-derived LP allele each (0.042 [0–0.122] frequency of each -13907:G, -13915:G, and -14009:G). A previous study has observed similar frequencies of -13915:G (0.00) and -13907:G (0.08) in the Danagla (Ingram et al. 2007). The Halfawieen only carry derived alleles of -13915 (0.111 [0–0.256]), concurrent with previous results (Hassan et al. 2016), and the Mahas have one individual with heterozygous state of -14009:G (0.036 [0–0.104]). The -13915:G allele was not observed in the Mahas in this study but has been previously observed (0.038–0.167, Enattah et al. 2008; Hassan et al. 2016). The Nubians and Sudanese Arab populations have similar levels of Middle Eastern admixture (Hollfelder et al. 2017); however, the Nubians show lower frequencies of the LP-associated alleles. The genetic differentiation of the LP-associated alleles between Nubians and central Sudanese Arabs is higher than 0.05 in three of the nine pairwise comparisons, when measuring a Nubian versus the Bataheen population. The Bataheen also show differentiation in the LP-associated alleles to the Gaalien (*F*_ST_ > 0.05). The Bataheen show the highest frequencies of LP-associated alleles and have the highest predicted LP phenotype of the Nubian and Sudanese Arab populations (table 2). Assuming that the non-African admixture into all Sudanese Arab and Nubian populations occurred during the same event, it is likely that the high occurrence of the putative LP phenotype is due to adaptive gene flow in the camel-breeding Bataheen, consistent with previous observations of a selective advantage of LP in dairy-farming populations.

#### The Nilotic Populations

No LP-associated alleles were found in the Nilotic populations of South Sudan (Shilluk, Dinka, Nuer, and Baria). Due to the close proximity of South Sudanese populations to East Africa, it is surprising that there is no evidence of the derived -14010:C allele in the Nilotic populations. This allele occurs in Nilotic Tanzanians and Kenyans, where it is significantly associated with LP (Tishkoff et al. 2007). The lack of LP-associated alleles in the agro-pastoralist Nilotic populations has been observed before (Tishkoff et al. 2007; Hassan et al. 2016) despite the intermediate prevalence of lactose digesters (>20%) in tested Nilotic populations (fig. 1*B*) (Bayoumi et al. 1981, 1982; Tishkoff et al. 2007). In an early study of lactose digesters in Sudan (Bayoumi et al. 1981), the Nuba and the Messiria also showed higher LP phenotypes than predicted in this study. These populations are genetically close to the Nilotic populations (Hollfelder et al. 2017) and LP might be driven by the same unknown mechanism/mutations as in the Nilotes. Figure 1*B* also shows that the observed frequencies of LP-associated alleles in some Sudanese Arab and Nubian populations cannot explain previous observations of the LP phenotype (Bayoumi et al. 1981). This difference might be caused by unknown LP variants or possibly adaption in the gut microbiome.

### Additional Observed Polymorphisms

Additional SNPs were found within the 316-bp region that have not been associated with LP (table 1). The -13913:C>T (rs41456145) polymorphism was found in heterozygous state in one Mahas and one Copt individual (allele frequencies: 0.0357 and 0.0454). Although this SNP is inside the Oct-1 binding site (Ingram et al. 2007), it does not appear to have an effect on LP (Jones et al. 2013). This SNP has previously been found in the Gaalien of Sudan and Fulani of Cameroon (Ingram et al. 2007), Khoe–San populations (Breton et al. 2014; Macholdt et al. 2014; Ranciaro et al. 2014), and Ethiopian populations (Jones et al. 2013). One Bataheen individual was found to be heterozygous for -14011:G>A (rs4988233) (0.0556). This SNP has been shown to influence promoter activity in vitro (Liebert et al. 2016) and has previously been observed in European and Middle Eastern populations (Lember et al. 2006; Liebert et al. 2016), Bantu-speaking populations of southern Africa (Macholdt et al. 2014), as well as Ethiopian populations (Jones et al. 2013). The allele -14107:T (rs574071884) was found in one instance in a Beni Amer individual (0.03125). This SNP has previously been found in Xhosa and Ghana populations (Torniainen et al. 2009), the Fulani of Mali (Lokki et al. 2011), Shuwa Arabs of Chad (Priehodová et al. 2014), and Bantu populations of Southern Africa (Macholdt et al. 2014). One allele of -14108:A (rs56150605) has been found in the Danagla. This allele has previously been encountered in the Gaalien of Sudan (Enattah et al. 2008).

### Haplotype Structure and Selection Scans

**Fig. 2:**
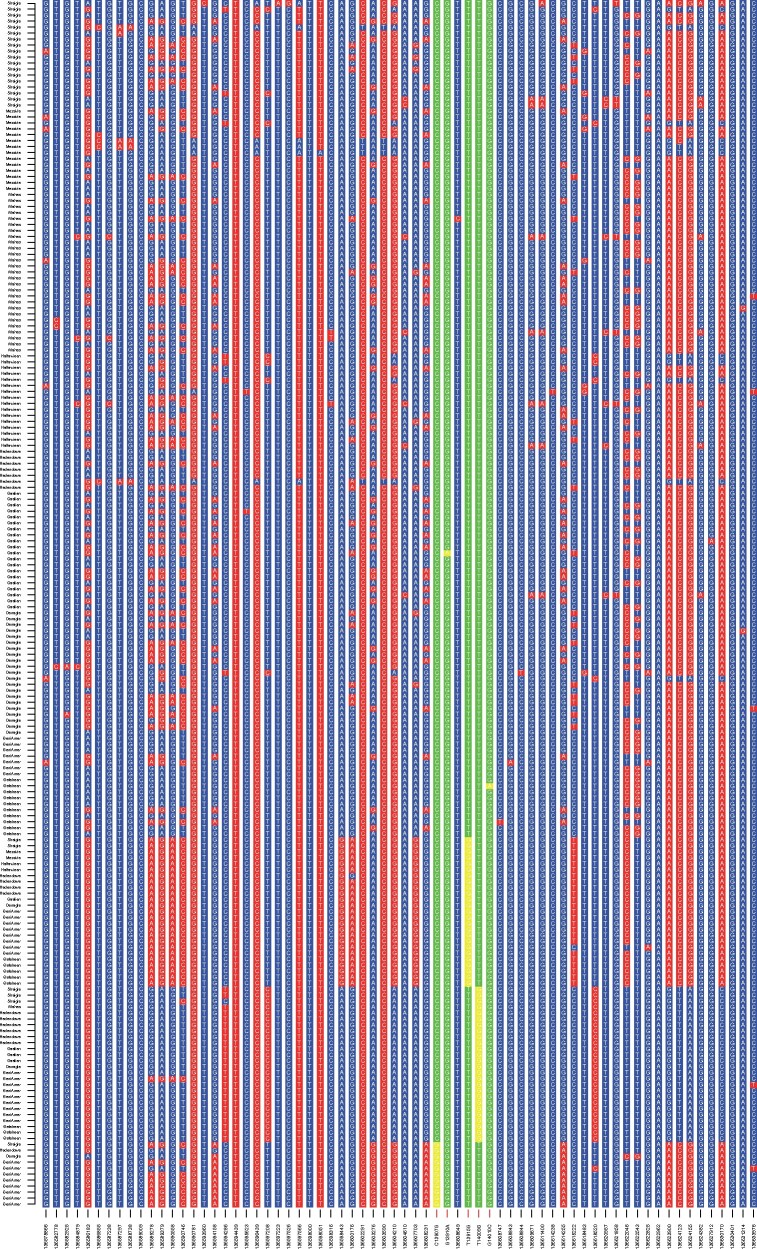
Haplotype background. Only polymorphic SNPs are shown over an extension of 30 kb in either direction of the LP-associated alleles in the populations that carry LP-associated alleles (Beja, Sudanese Arab, Nubian). Each line represents one chromosome. Lactase-associated positions are highlighted in green/yellow, where yellow is a derived allele. The *x* axis shows the bp-positions of the SNPs in GRCh37.p13, with the exception of the positions that have been associated with LP. Here, the position upstream of *LCT* is shown along with the ancestral and derived LP-associated allele. As the figure is showing the forward strand, alleles on position -13910 are complementary.

**Fig. 3:**
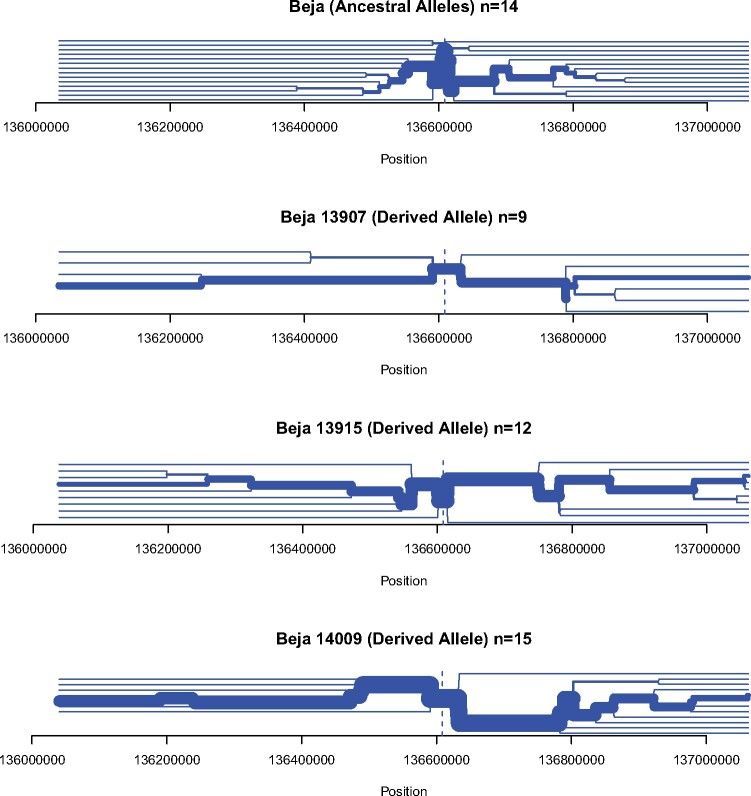
Bifurcation plots for the Beja populations. For each plot, *n* denotes the number of investigated alleles. The thickness of the line corresponds to the number of individuals sharing the haplotype. The topmost plot is centered around position -13907 and contains only haplotypes that have none of the derived LP-associated alleles.

We created a plot showing the allelic state of each SNP in the populations containing the three LP-associated alleles found in moderate frequencies in the investigated populations: -13907, -13915, and -14009 (fig. 2). As observed before (Tishkoff et al. 2007), the LP-associated SNPs are found in distinct haplotype blocks and have therefore evolved independently. This is also observed in the haplotype network (supplementary fig. S2, Supplementary Material online). Furthermore, bifurcation plots were created to visualize the extension of the haplotypes surrounding the LP-associated alleles (supplementary figs. S3–S5, Supplementary Material online). They show large extensions in the Beja population, who carry the highest number of LP-associated alleles (fig. 3). These plots might, however, overrepresent haplotypes due to allelic dropout in the investigated samples (see Material and Methods, supplementary fig. S6, Supplementary Material online) (Hollfelder et al. 2017). This loss of alleles during whole-genome amplification causes long spurious runs of homozygosity and might be the cause for a particular long run of homozygosity around the position of the LP alleles in a Hadendowa individual, who is homozygous for -14009:G (fig. 3 and supplementary fig. S6, Supplementary Material online).

**Fig. 4:**
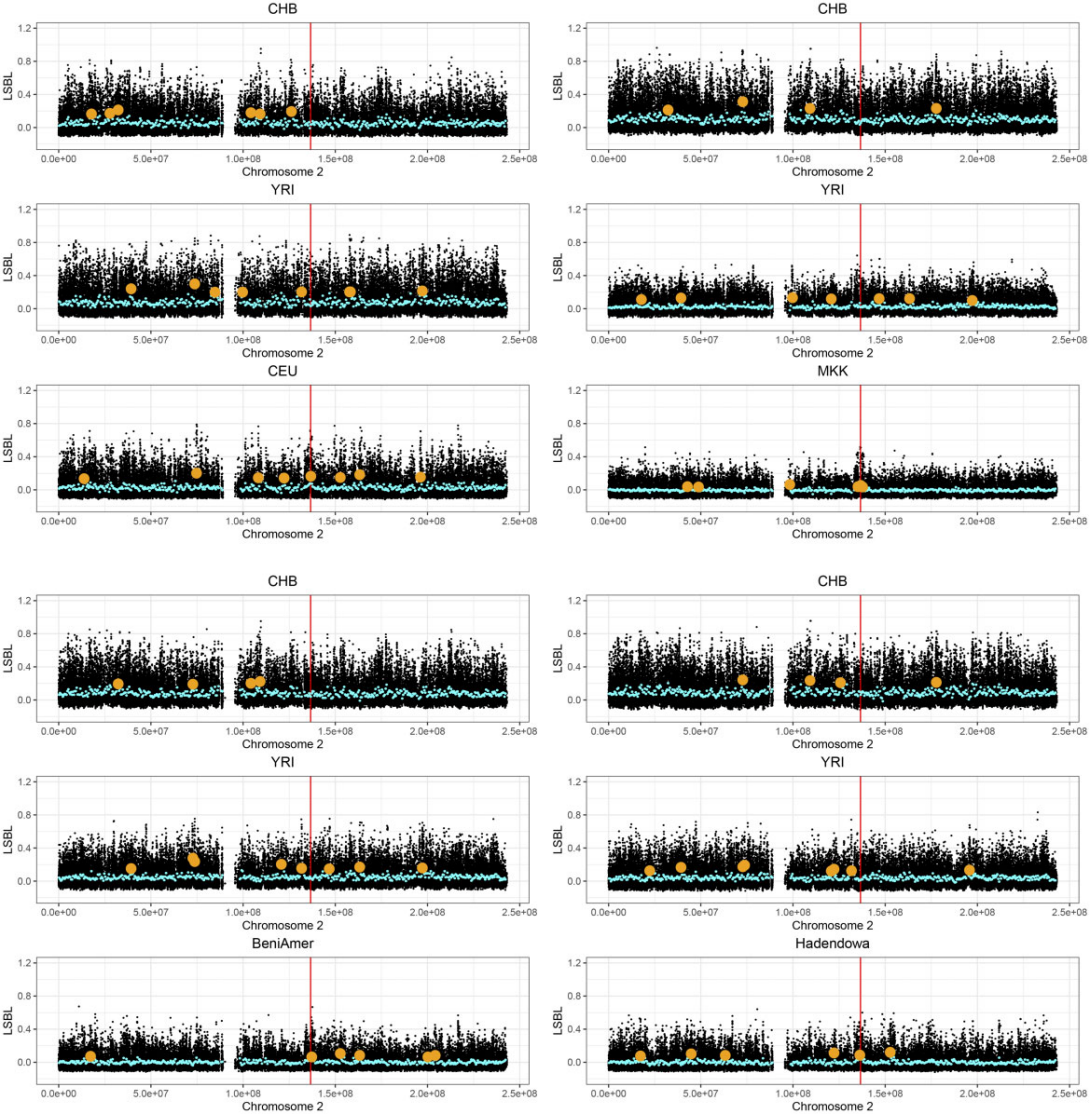
LSBL result for CEU, MKK, and the Beja populations. A group of three plots shows the population-specific branch length for the combination CHB, YRI, and X, where X is the population on the third plot of the group. The blue points indicate the means of 500 kb windows, the larger orange points show windows that deviate from the mean by more than three standard deviations (*P* < 0.0027). The red vertical line shows the position of -13910:C>T.

To investigate whether the high frequencies of putative lactose digesters are the result of a selection event, we performed selection scans. We computed the LSBL statistic (Shriver et al. 2004) across chromosome 2 and for each SASS population, as well as the MKK and CEU populations of the 1000 Genomes Project data set (1000 Genomes Project Consortium 2015), to search for signals of positive selection (fig. 4 and supplementary figs. S7–S11, Supplementary Material online). The area around the LP-associated polymorphisms is a clear outlier in MKK and CEU (*P *=* *0.0014 and *P *=* *0.0005), which have previously been shown to be subjected to strong positive selection (Bersaglieri et al. 2004; Schlebusch et al. 2013). Both Beja populations show increased LSBL signals in one of the neighboring windows (*P *=* *0.0467 in the Beni Amer and *P *=* *0.0032 in the Hadendowa). Two other regions on chromosome 2 are distinguished from the comparative populations in the LSBL analysis and affect more than four populations (supplementary table S1, Supplementary Material online). To further investigate the signal seen in the Beja populations in the LSBL analysis, XP-CLR (Chen et al. 2010) was performed on the Beja populations separately and combined using the Dinka as a reference. For comparison, we also performed the test on the MKK. XP-CLR is robust to ascertainment bias and can be used to detect soft sweeps. Although we observe a clear peak around the LP-associated allele position in the MKK, no signal is observed in the Beja (supplementary fig. S12, Supplementary Material online). It is unclear whether the negative results is due to a lack of power given the number of causal variants on different haplotypic backgrounds or whether it can be interpreted as absence of selection.

The high frequency of individuals carrying at least one LP-associated allele in the Beja populations points to an increasing fitness for the LP phenotype. The high diversity of LP-associated alleles makes it difficult to observe strong selection signals, as selection would act on all three variants, and the overall outcome might resemble a soft sweep where several variants are adaptive.

## Conclusion

LP-associated alleles from Europe (-13910:T) and East Africa (-14010:C) have been used to track migration patterns of African populations (Myles et al. 2005; Enattah et al. 2007; Coelho et al. 2009; Breton et al. 2014; Ranciaro et al. 2014; Ben Halima et al. 2017). Sudanese populations have been shown to be recipients of non-African gene flow, likely from a Middle Eastern source (Hollfelder et al. 2017). The absence of the European and East African LP alleles (-13910:T and -14010:C) suggests negligible amounts of gene flow from LP populations from these regions into the populations of Sudan and South Sudan, whereas the occurrence of the allele associated with LP in the Middle East (-13915:G) is consistent with gene flow from the Middle East into Sudan.

Even though this study investigated a range of Nilotic populations, no LP-associated SNPs were detected in these agropastoralist populations. However, Nilotic agropastoralist have been shown to be able to digest milk in hydrogen breath tests and lactose tolerance tests (Bayoumi et al. 1981, 1982; Tishkoff et al. 2007). This observation is intriguing, and future studies on Nilotic populations might reveal more about the underlying biology of LP in these populations. We also note that some other Sudanese populations present modest frequencies of the LP-associated alleles in comparison with observed levels of lactose digesters. The traditionally pastoral Beja people have been shown to have one of the highest level of lactose digesters in the world (Bayoumi et al. 1981; Holden and Mace 1997; Tishkoff et al. 2007). Both -13907:G and -14009:G appear at their highest frequency in the Beja and are most prevalent in the surrounding area, possibly pointing a point of origin. However, these SNPs have not been widely investigated in North Africa (supplementary fig. S13, Supplementary Material online) and outside of Africa (Liebert et al. 2017). Another LP-associated SNP, -13915:G also appears at high frequency in the Beja populations. The three alleles found in the Beja populations are on different haplotype backgrounds driving the frequency of putative lactose digesters to the highest seen in the area (table 2 and fig. 1*A*). There is a clear extension of the haplotypes surrounding the derived alleles of the SNPs associated with LP (fig. 3). There is also an increase in LSBL values close to the LP-associated region, yet the signal is not significant in XP-CLR tests. Positive selection in the Beja populations for LP-associated alleles -13907:G and -13915:G has been suggested previously (Ranciaro et al. 2014), and the high prevalence of the LP phenotype in the Beja populations still suggests that there is, or has been, an adaptation to digest milk. Further studies are needed to clarify the process that drove the Beja to such high frequencies and to get a better understanding of the emergence and history of -13907:G and -14009:G variants.

## Materials and Methods

A total of 221 individuals from 18 Sudanese and South Sudanese populations were selected for sequencing. These individuals have previously been investigated using microsatellites (Babiker et al. 2011) and dense SNPs (Hollfelder et al. 2017). Subsistence strategy was determined during sampling, from conversations with researchers, and literatures (Bayoumi et al. 1981, 1982; Rone 2003; Deng 2010; Hassan et al. 2016). The individuals sampled in this study have given informed consent and the research was approved by the forensic labs ethic review panel of Sudan (No. G F E/52/B/1). A 316-bp region of intron 13 of the *MCM6* gene was targeted for sequencing, encompassing all variants associated with LP (-13907, -13910, -13915, -14009, and -14010). Primer sequences were obtained from Coelho et al. (2009). DNA was extracted from Whatman FTA cards using Whatman protocol BD09 and BD01. Polymerase chain reaction was performed using 0.625 U AmpliTaq Gold DNA Polymerase, 1× Gold Buffer, 0.5 mM dNTP mix, 2.5 mM MgCl_2_, and 0.2 µM of each primer per reaction in 30 cycles of 95 °C at 15 s, 55 °C at 30 s, and 72 °C at 45 s, with an initial deamination step of 10 min at 95 °C and a final elongation of 5 min at 72 °C. Sanger sequencing was performed at the Uppsala Genome Center, which is part of the Swedish National Genomics Infrastructure. The computations were performed on a high performance compute cluster at Uppsala’s Multidisciplinary Center for Advanced Computational Science (UPPMAX).

The obtained electropherograms were visually checked using GeneStudio and aligned to hg19 using MEGA7 (Kumar et al. 2016). Of the 221 individuals sequenced, 203 individuals gave successful sequencing results. All polymorphic sites were covered by concordant forward and reverse strands except for two individuals (one from each the Shaigia and the Bataheen populations) who had a successful result only with the forward primer. All polymorphism peaks were unambiguous.

The standard error (SE) for the allele frequencies was calculated using a following binomial approximation:

$$\text{SE}=\sqrt{\frac{p(1-p)}{2N}},$$

where *p* is the allele frequency of the derived allele and *N* is the number of samples. A confidence interval (1.96×SE) is given in squared brackets after allele frequencies in text. A lactase persistent phenotype frequency was calculated for each population by dividing the number of samples that carry at least one LP-associated allele by the total number of individuals per population.

### Phasing and Imputation to Analyze Haplotype Structure

The genotyping results were added to 323,726 additional SNPs from chromosome 2, obtained from a filtered data set of 3.9 million SNPs, typed on an Illumina HumanOmni5M Exome SNP array in a previous study (Hollfelder et al. 2017). This combined data set was phased and missing data were imputed using fastPHASE version 1.4.0 (Scheet and Stephens 2006). The number of haplotype clusters was set to 25, with 25 runs of the EM algorithm. The number of haplotypes sampled from the posterior distribution obtained from a particular random start of the EM algorithm was set to 100. We used the phase information to create a visualization of the haplotypes surrounding the LP control region (fig. 2). The R-package “rehh” (Gautier and Vitalis 2012) was used to create bifurcation plots visualizing the haplotype structure surrounding the LP-associated alleles (fig. 3 and supplementary figs. S8–S10, Supplementary Material online). The haplotype network was created with the phylogenetic network software by fluxus-engineering using a Median-Joining network (Bandelt et al. 1999).

### Selection Scans

Whether the region surrounding the LP-associated alleles show signals of selection was investigated using LSBL (Shriver et al. 2004) and XP-CLR (Chen et al. 2010). LSBL estimates the branch length per locus by comparing pairwise *F*_ST_ values of three populations. This allows to detect in which of the three populations the genetic differentiation took place. XP-CLR highlights regions in the genome where rapid allele frequency change occurred as assessed by the size of the affected region.

LSBL was calculated on the SASS populations from the data set of Hollfelder et al. (2017) as well as genotyped populations from the 1000 Genomes Project (1000 Genomes Project Consortium 2015) (Yoruba in Ibadan, Nigeria [YRI], Han Chinese in Beijing, China [CHB], MKK, and Utah residents with northern and western CEU ancestry). Each of the 1000 Genomes project populations was downsampled to a sample size of 16 to match the sample size of the data generated in this study. The data set of Hollfelder et al. (2017) experienced a degree of allelic dropout, which excludes the possibility of selection scans using haplotype-based methods for this data set. It was, however, shown that *F*_ST_ estimates on this diploid data set correlate strongly with a randomly haploidized version of the data set, therefore, measures such as LSBL can be used safely on the fully diploid data set (Hollfelder et al. 2017, SI).

We calculated Weir and Cockerham’s *F*_ST_ as implemented in plink v1.90 (Chang et al. 2015). LSBL was calculated for each locus on the SASS populations using two comparative non-LP populations (YRI and CHB, 1000 Genomes Project Consortium 2015), one African and one non-African to account for admixture in the SASS populations.

$${\text{LSBL}}_{\text{pop}}=\frac{F_{\text{ST}}^{\text{YRI},\;\text{pop}}+F_{\text{ST}}^{\text{CHB},\;\text{pop}}-F_{\text{ST}}^{\text{YRI},\;\text{CHB}}}{2},$$

where pop is the test population. LSBL is calculated for each of the three combined populations. All SASS populations were tested, as well as MKK and CEU, which have been subjected to strong selection in the genomic region of the LP-associated alleles (Bersaglieri et al. 2004; Schlebusch et al. 2013). We computed the mean LSBL in nonoverlapping 500 kb windows containing at least 50 SNPs and highlighted areas that are more than three standard deviations higher than the mean (fig. 4 and supplementary figs. S2–S6, Supplementary Material online). We calculated *P* values from the *Z* score and corrected them for multiple testing using the Benjamini–Hochberg correction (Benjamini and Hochberg 1995). The window containing the LP-associated alleles in MKK and CEU were significant with this cutoff (*P *<* *0.005), showing that we have enough power to detect increased LSBL with the given sample sizes. A control was performed where negative *F*_ST_ estimates were exchanged to 0 (Hider et al. 2013). The treatment of negative *F*_ST_ estimates did not have an impact on the results (supplementary table S1, Supplementary Material online).

XP-CLR was performed on the same data set as used for LSBL. The combined genetic map from the 1000 Genomes Project (Sudmant et al. 2015) was used to infer and interpolate the genetic distance of each SNP in our database. We investigated the MKK, the BeniAmer, the Hadendowa, as well as a combination of the two Beja populations as objective populations, using the Dinka as a reference population. XP-CLR was performed on phased data with a grid size of 2 kb, 0.2 cM window size, a maximum of 100 SNPs per window, and a correlation level of 0.95.

## Supplementary Material

Supplementary data are available at *Genome Biology and Evolution* online.

## Supplementary Material

evab065_Supplementary_Data
