# Supplementary material for: The Genetic Variation of Lactase Persistence Alleles in Sudan and South Sudan
Source: Genome Biol Evol. 2021 Mar 24;13(5):evab065. doi: 10.1093/gbe/evab065 (PMC8175049; doi:10.1093/gbe/evab065)
Supplement: evab065_Supplementary_Data [file evab065_supplementary_data.pdf]

Supplementary Figures and Tables - The genetic variation of lactase persistence alleles in northeast Africa

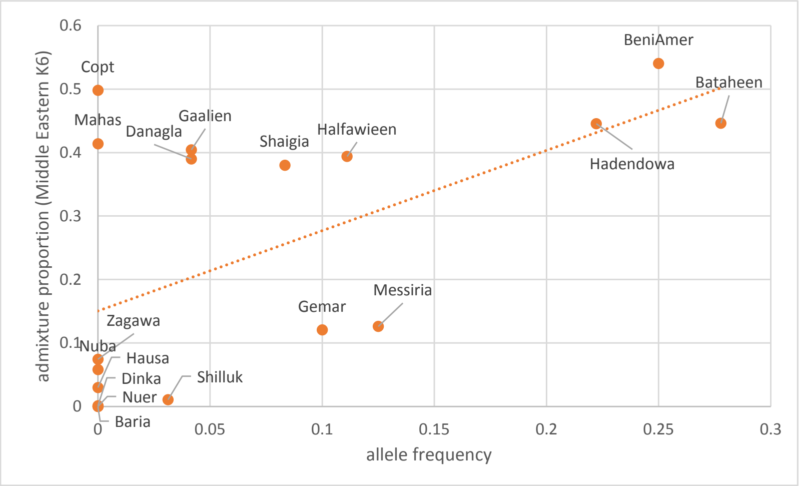

Figure S1: Comparison of allele frequency of -13915:G to the Middle Eastern ancestry component assuming 6 clusters in an ADMIXTURE analyses with worldwide populations [Hollfelder et al., 2017, Fig. S3].

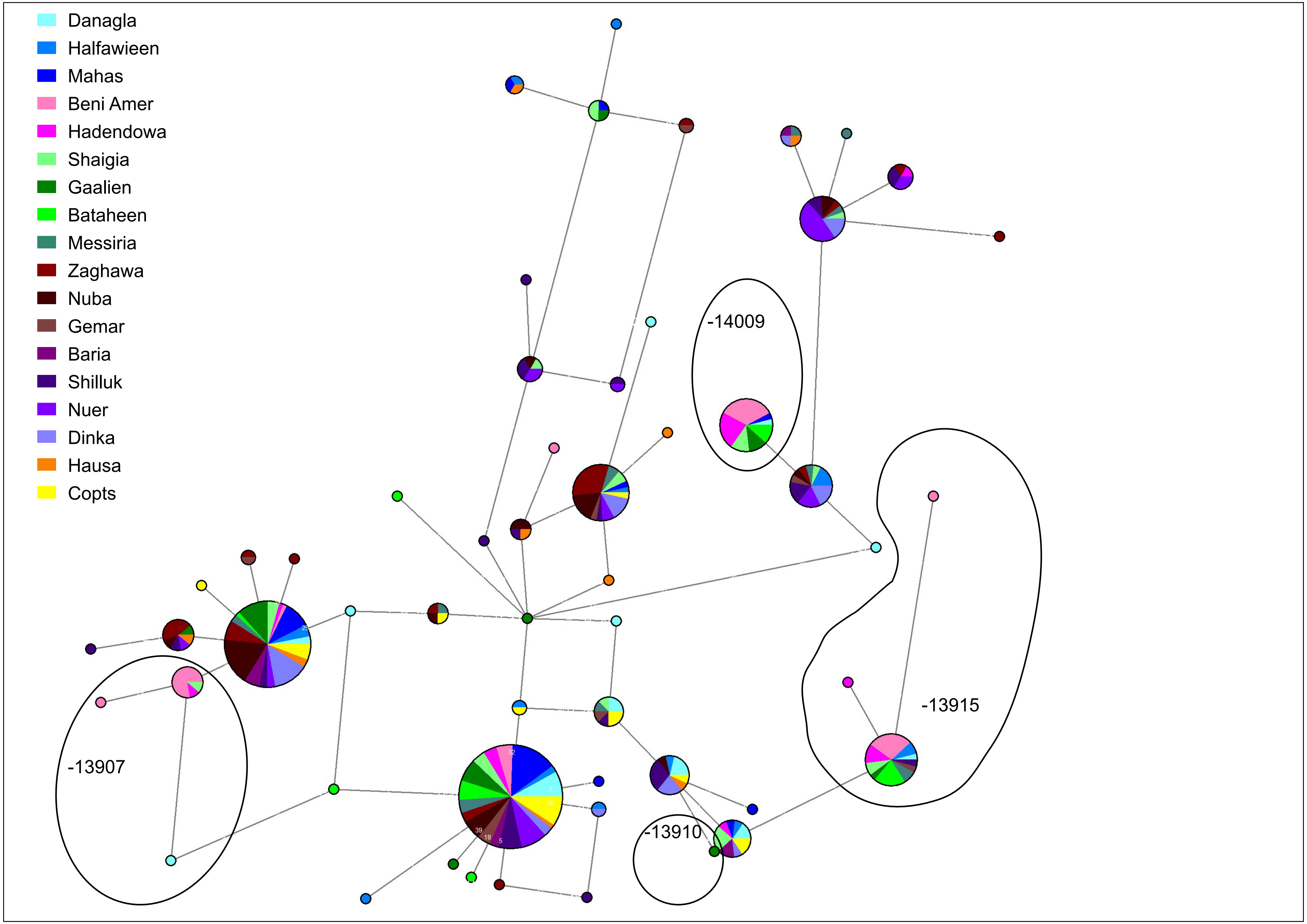

Figure S2: Haplotype network of polymorphic SNPs surrounding the Lactase SNPs, 10kb downstream and 20kb upstream.

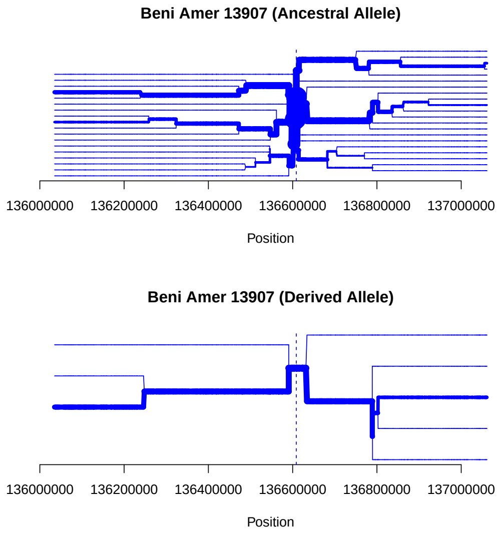

Figure S3: Haplotype structure around -13907

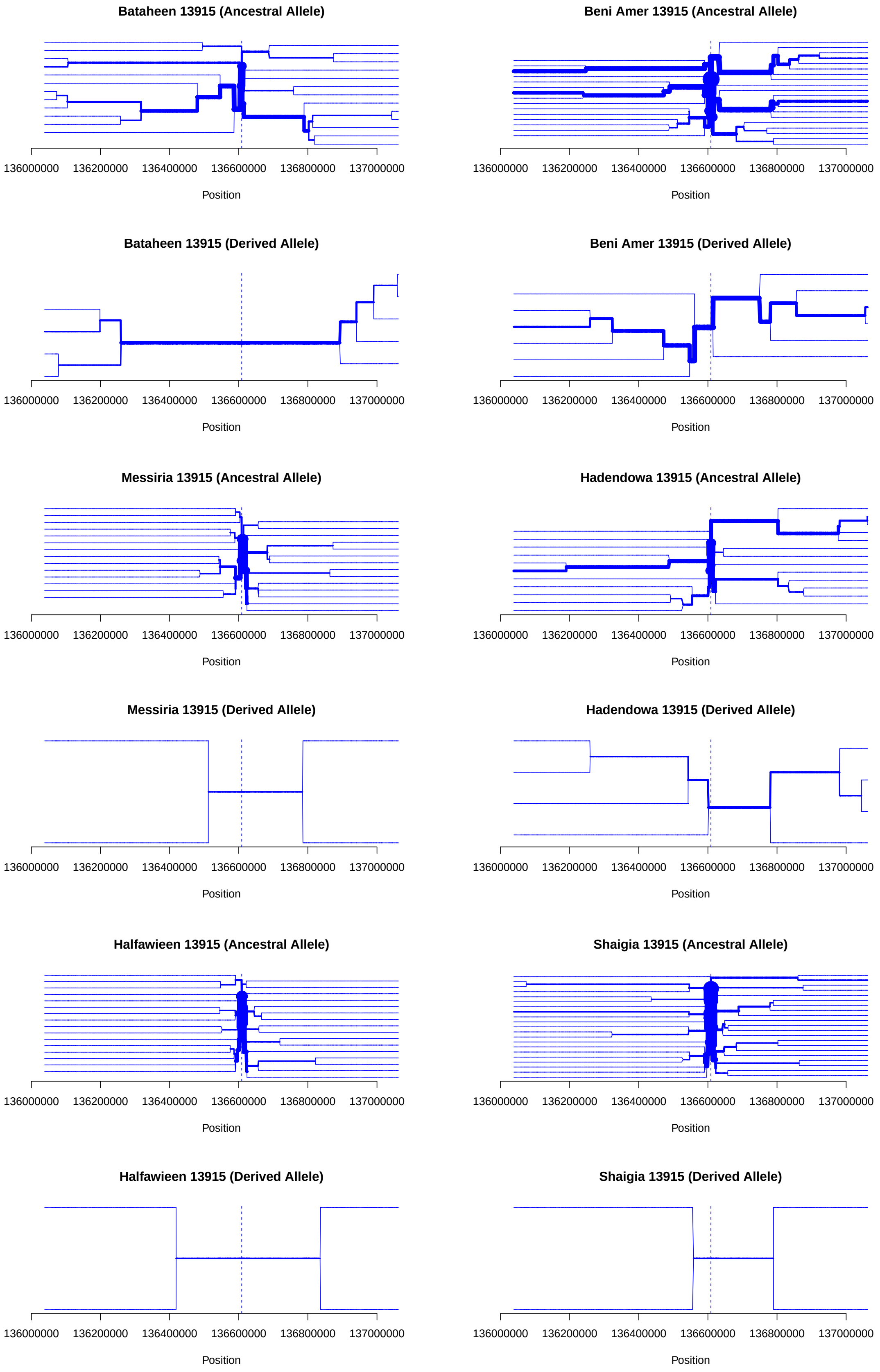

Figure S4: Haplotype structure around -13915

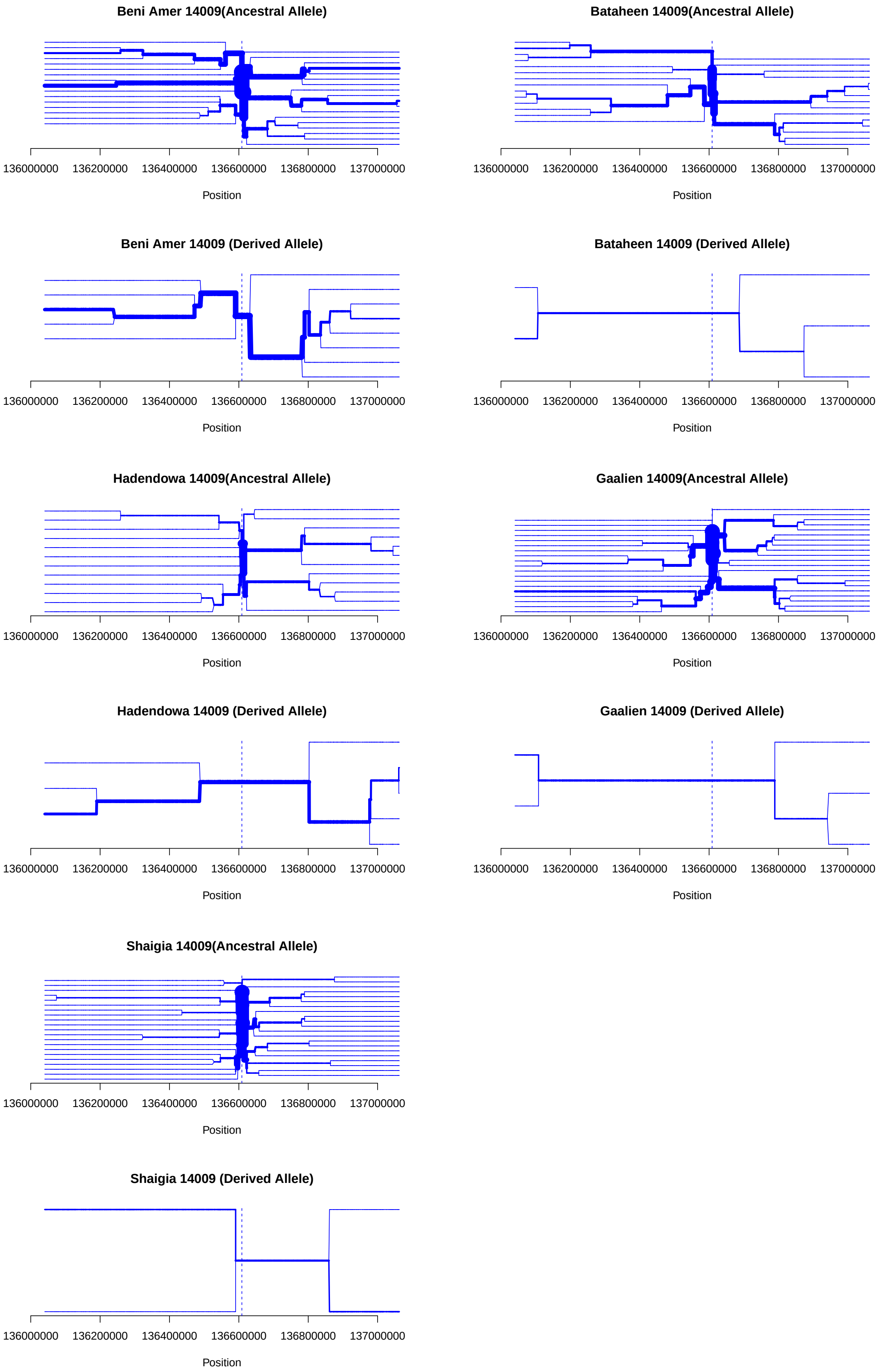

Figure S5: Haplotype structure around -14009.

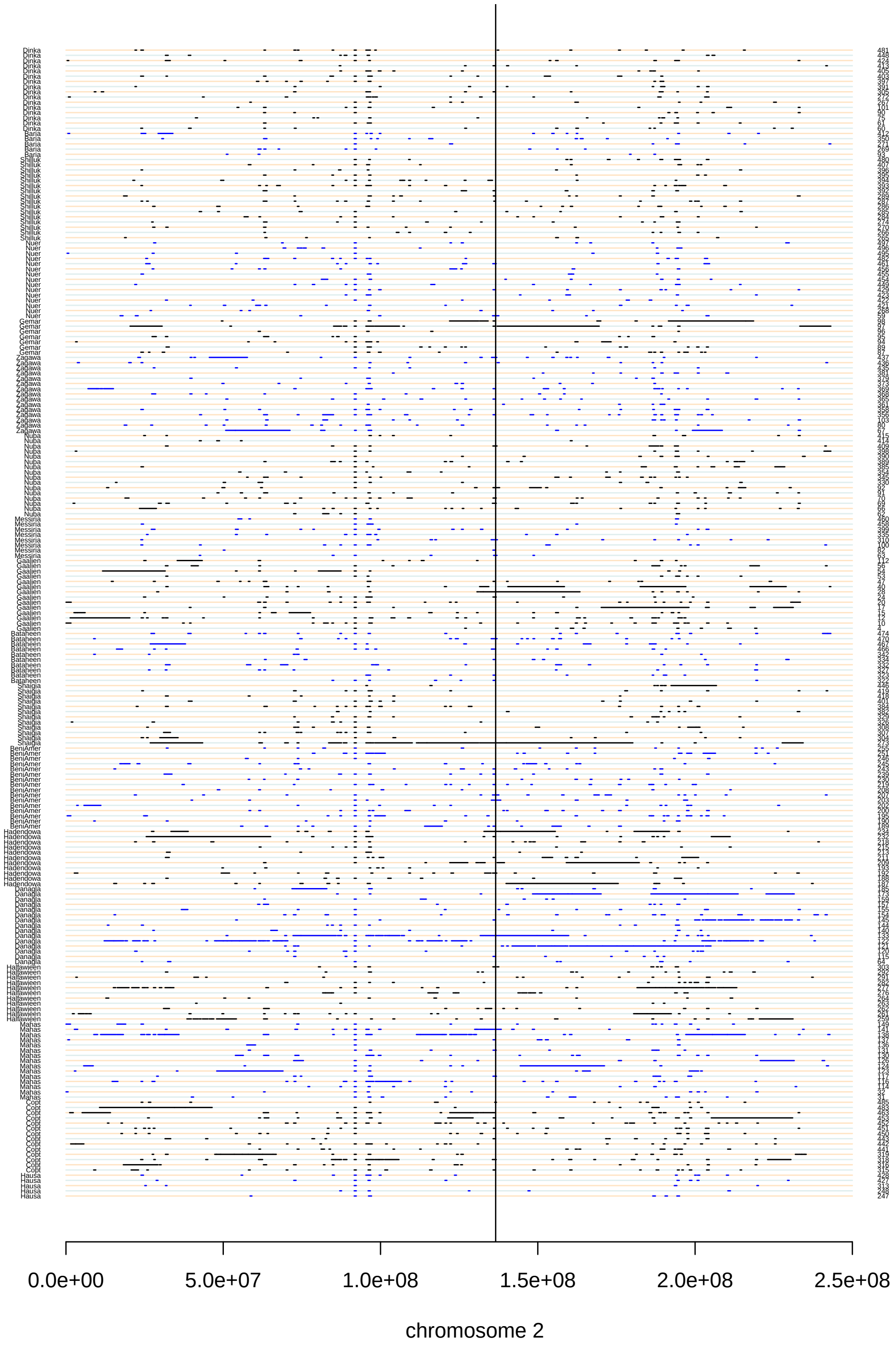

Figure S6: Runs of Homozygosity on chromosome 2. The X-axis shows the chromosomal position, the vertical black bar shows the position of 13910:C>T. The Y-axis on the left shows the population, the right the individual ID.

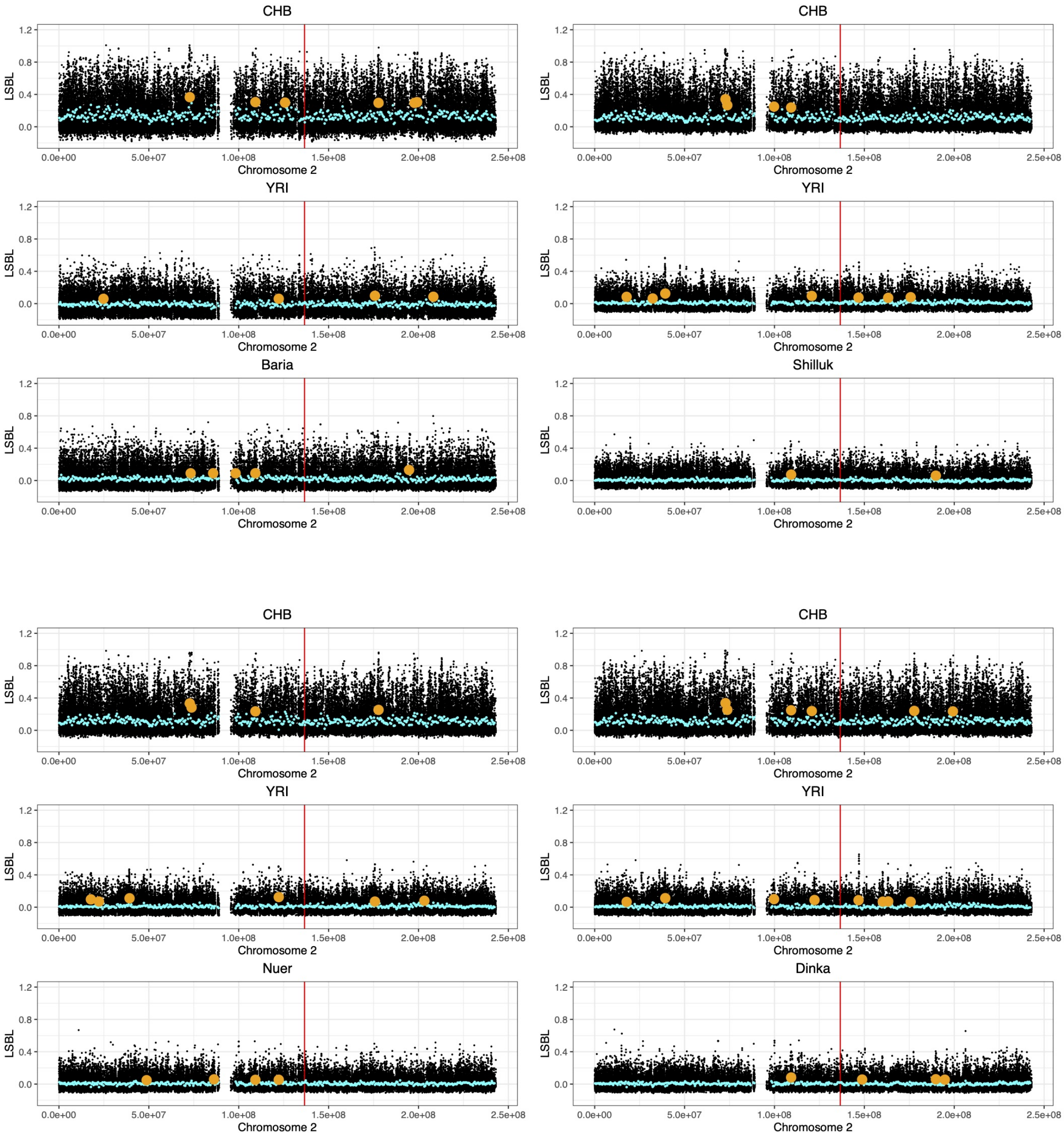

Figure S7: LSBL of the Nilotes. A group of three plots shows the population specific branch length for the combination CHB, YRI and X, where X is the population on the third plot of the group. The blue points indicate the means of 500kb windows, the larger orange points show windows that deviate from the mean by more than 3 standard deviations. The red vertical line shows the position of -13910:C>T.

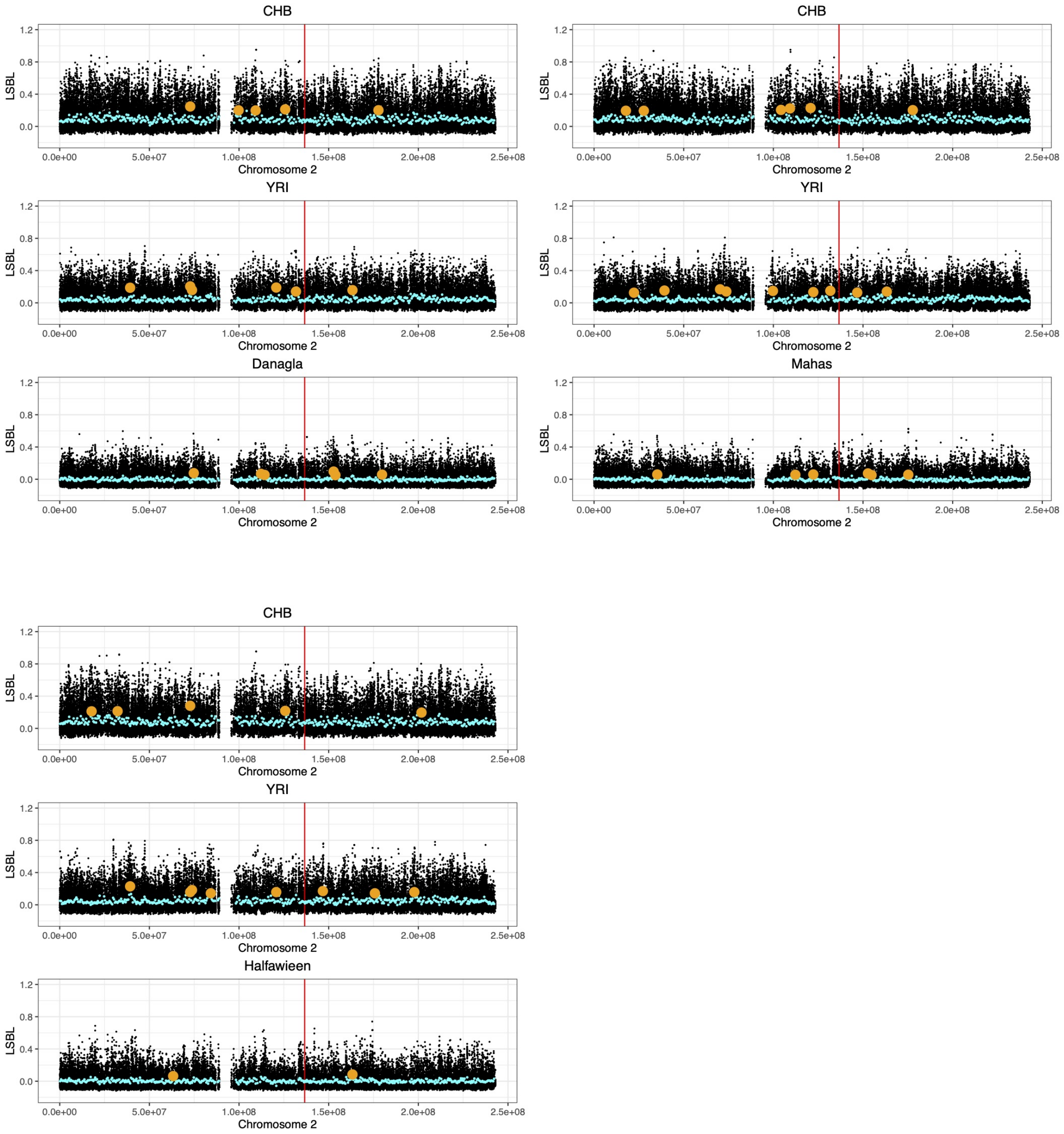

Figure S8: LSBL of the Nubians. A group of three plots shows the population specific branch length for the combination CHB, YRI and X, where X is the population on the third plot of the group. The blue points indicate the means of 500kb windows, the larger orange points show windows that deviate from the mean by more than 3 standard deviations. The red vertical line shows the position of 13910:C>T.

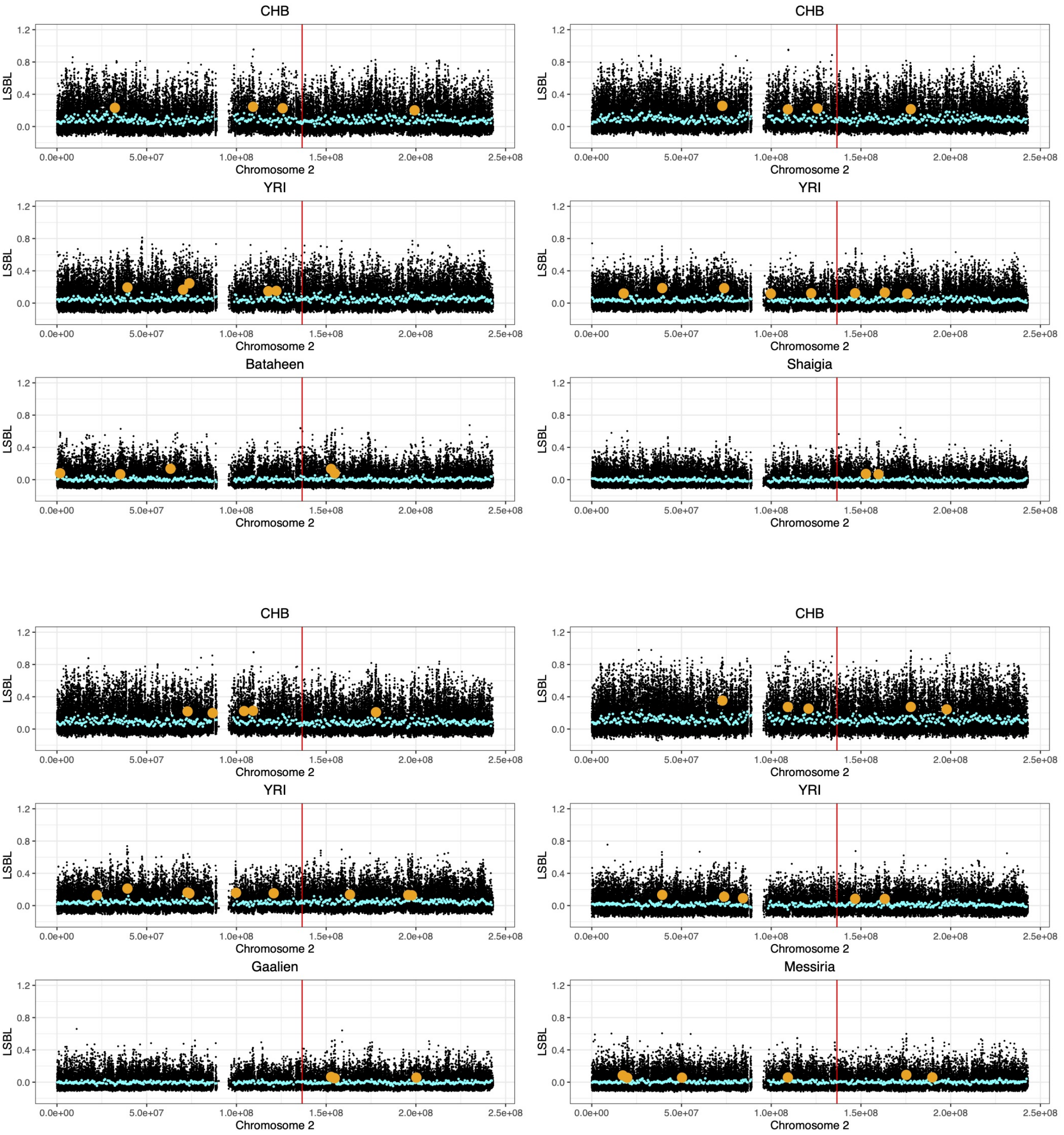

Figure S9: LSBL of the Arab populations. A group of three plots shows the population specific branch length for the combination CHB, YRI and X, where X is the population on the third plot of the group. The blue points indicate the means of 500kb windows, the larger orange points show windows that deviate from the mean by more than 3 standard deviations. The red vertical line shows the position of 13910:C>T.

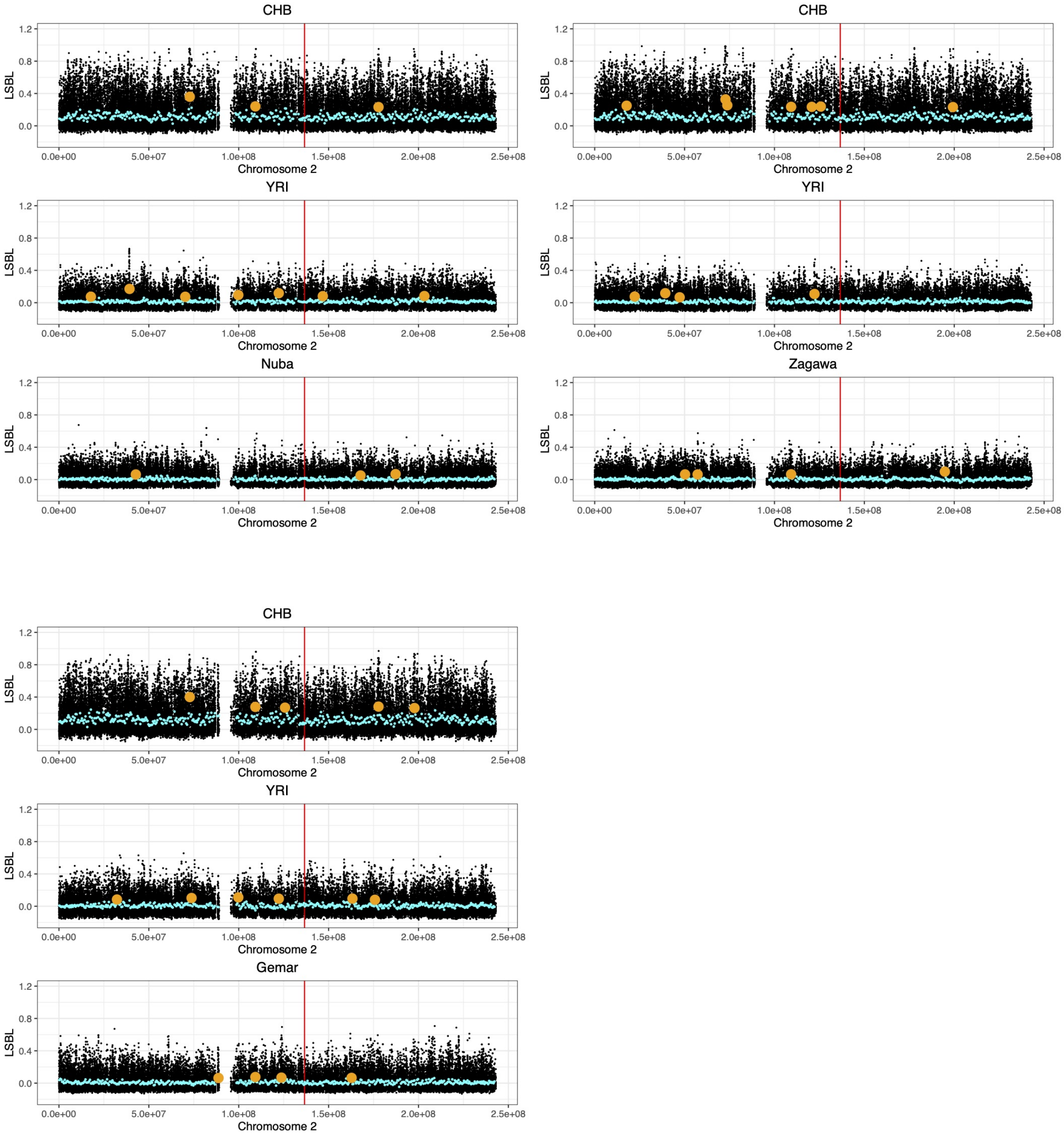

Figure S10: LSBL of the Darfurian/Kordofonanian populations. A group of three plots shows the population specific branch length for the combination CHB, YRI and X, where X is the population on the third plot of the group. The blue points indicate the means of 500kb windows, the larger orange points show windows that deviate from the mean by more than 3 standard deviations. The red vertical line shows the position of 13910:C>T.

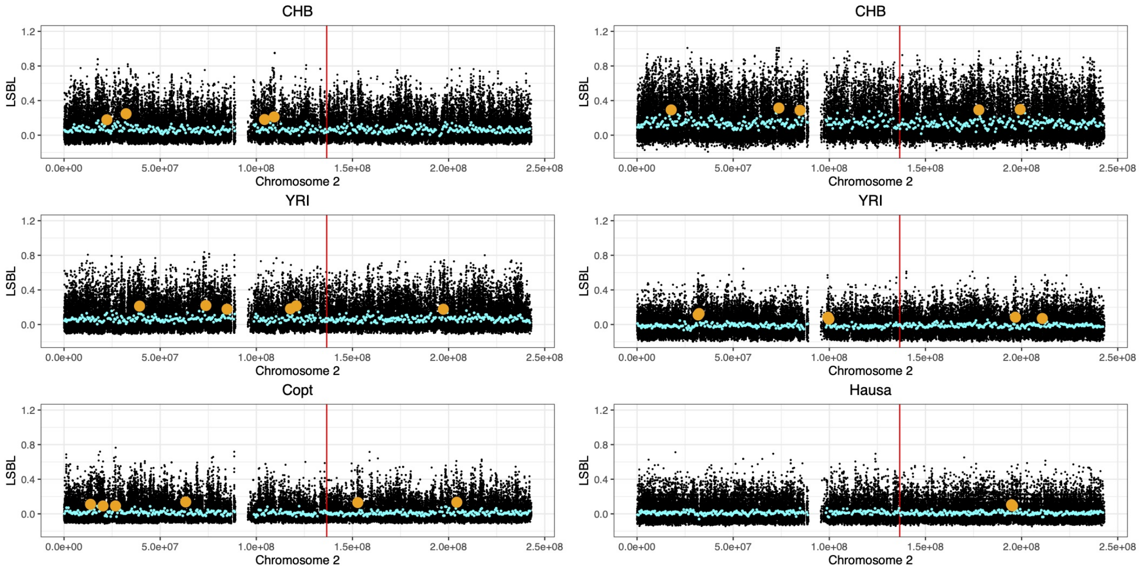

Figure S11: LSBL of the migrant populations. A group of three plots shows the population specific branch length for the combination CHB, YRI and X, where X is the population on the third plot of the group. The blue points indicate the means of 500kb windows, the larger orange points show windows that deviate from the mean by more than 3 standard deviations. The red vertical line shows the position of 13910:C>T.

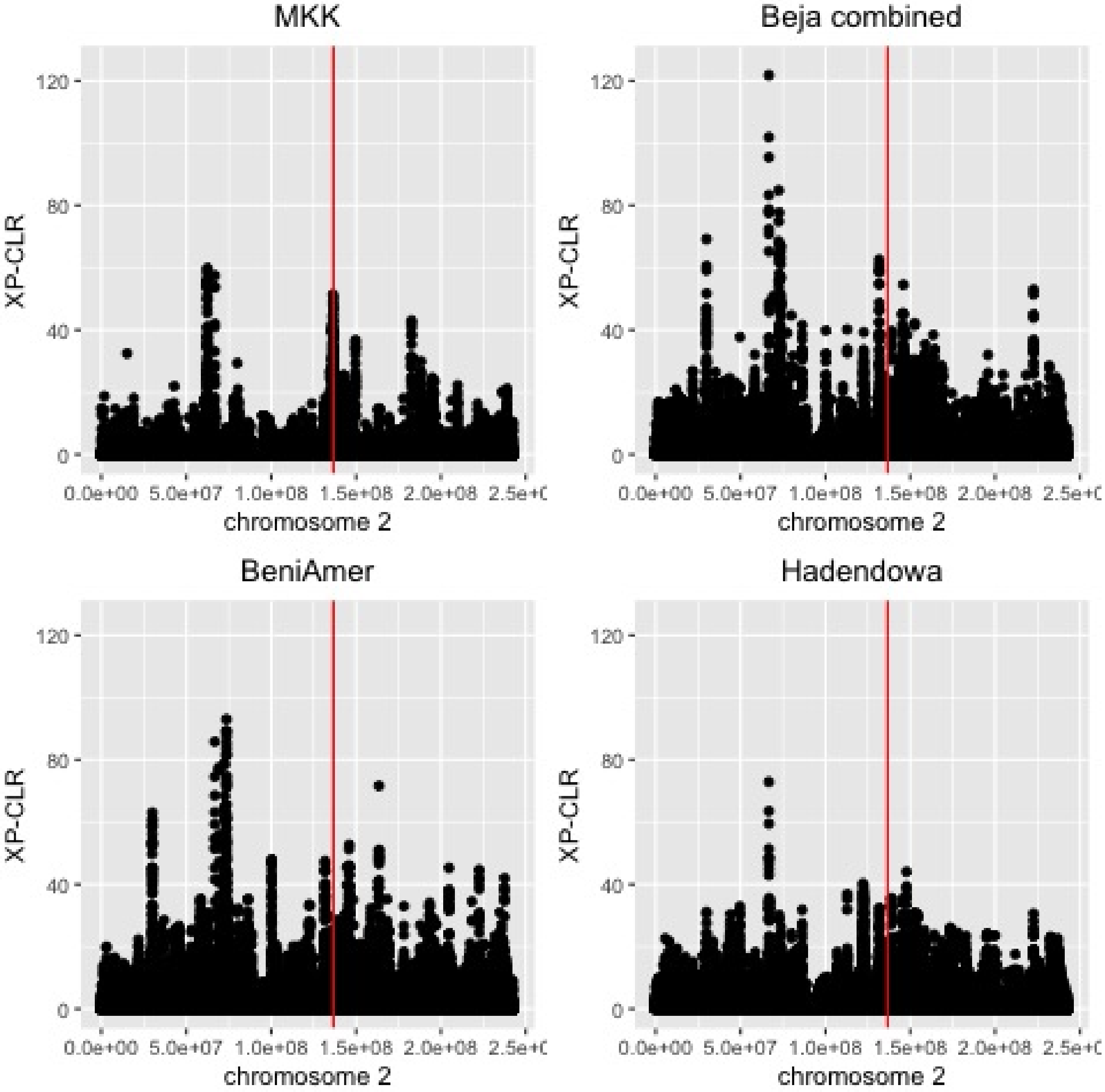

Figure S12: XP-CLR performed on MKK, BeniAmer, Hadendowa and both Beja populations combined using Dinka as reference. The red vertical line marks the position of -13910:C>T.

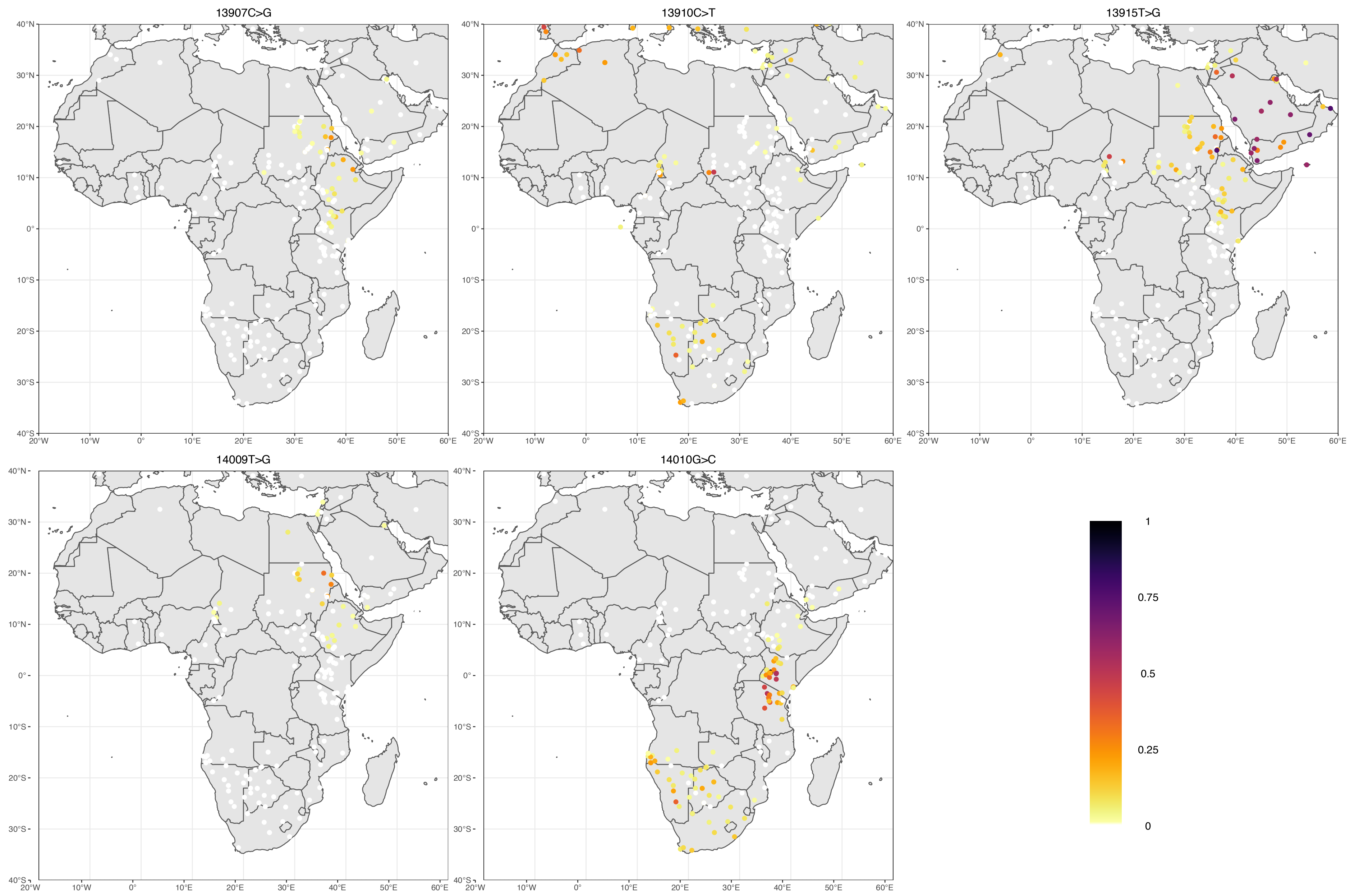

Figure S13: Allele frequency distribution of the derived alleles of the investigated SNPs in Africa. The white color signifies an allele frequency of zero. Data from this study and Liebert et al. [2017]

Table S1: Regions that have significantly long LSBL. Region that includes the LP-associated alleles is marked red.

500 kb window	both F_{ST} datasets	neg. F_{ST} allowed	neg. F_{ST} converted to 0
1518677-2018677	Bataheen		
13518701-14018701	CEU, Copt		
17018708-17518708	Beni Amer, Hadendowa, Messiria		
19518713-20018713	Messiria		
20018714-20518714	Copt		
26518727-27018727	Copt		
35018744-35518744	Bataheen, Mahas		
42518759-43018759	MKK, Nuba		
44518763-45018763	Hadendowa		
48518771-49018771		Nuer, MKK	
50018774-50518774	Zaghawa	Messiria	
57018788-57518788	Zaghawa		
63018800-63518800	Bataheen, Copt, Hadendowa, Halfawieen		
73018820-73518820	Baria		
74518823-75018823	CEU, Danagla		
84518843-85018843			Shilluk
85518845-86018845	Baria		
86018846-86518846	Nuer		
88518851-89018851			Gemar
98018870-98518870	Baria, MKK		
108018890-108518890	CEU		
109018892-109518892	Dinka, Gemar, Messiria, Nuer, Shilluk, Zaghawa	Baria	
112018898-112518898	Danagla, Mahas		
114018902-114518902	Danagla		
122018918-122518918	CEU, Hadendowa, Mahas, Nuer		
123518921-124018921	Gemar		
135018944-135518944	MKK		
136018946-136518946	Hadendowa		
136518947-137018947	CEU, MKK		
137018948-137518948	Beni Amer		
137518949-138018949	MKK		
148518971-149018971	Dinka		
152518979-153018979	Bataheen, Beni Amer, CEU, Copt, Danagla, Gaalien, Hadendowa, Mahas, Shaigia		
154518983-155018983	Bataheen, Gaalien, Mahas		Hadendowa
159518993-160018993	Shaigia		
162518999-163018999	Gemar		
163019000-163519000	Beni Amer , CEU, Halfawieen		
167519009-168019009	Nuba		
175019024-175519024	Mahas, Messiria		
179519033-180019033	Danagla		
187019048-187519048	Nuba		
189519053-190019053	Dinka, Messiria, Shilluk		
194519063-195019063	Baria, Dinka, Hausa, Zaghawa		
195019064-195519064	Hausa		
196019066-196519066	CEU		
200019074-200519074	Beni Amer, Gaalien		
204019082-204519082	Beni Amer, Copt		

There are two regions that are distinguished in more than four populations. The first region (2:152518979-153018979) harbors the following genes: *NEB*, *ARL5A*, *CACNB4*, and *STAM2*. These Genes are associated with: nemaline myopathy 2, anxiety disorders, pancreatitis, waist circumference, lung neoplasms, altruism, body height, epilepsy, alopecia, hemoglobins, alcoholism, and chemo-therapy induced alopecia in breast cancer patients. The second region (2:109018892-109518892) contains the genes *GCC2* associated with child development disorders, *LIMS1*, *CCDC138*, and *RANBP2* which is associated with susceptibility to encephalopathy, and is downstream of *EDAR*, which is associated with hair and tooth morphology and ectodermal dysplasia.

References

N. Hollfelder, C. M. Schlebusch, T. Gu, H. Babiker, H. Y. Hassan, and M. Jakobsson. Northeast African genomic variation shaped by the continuity of indigenous groups and Eurasian migrations. *PLOS Genetics*, 13(8):1–17, 8 2017. ISSN 1553-7404. doi: 10.5061/dryad.bs06h. URL `http://dx.plos.org/10.1371/journal.pgen.1006976`.

A. Liebert, S. López, B. L. Jones, N. Montalva, P. Gerbault, W. Lau, M. G. Thomas, N. Bradman, N. Maniatis, and D. M. Swallow. World-wide distributions of lactase persistence alleles and the complex effects of recombination and selection. *Human Genetics*, 136(11-12):1445–1453, 11 2017. ISSN 0340-6717. doi: 10.1007/s00439-017-1847-y. URL `http://link.springer.com/10.1007/s00439-017-1847-y`.
